# Gibson Deletion: a novel application of isothermal in vitro recombination

**DOI:** 10.1186/s12575-018-0068-7

**Published:** 2018-01-19

**Authors:** Swara Kalva, Jef D. Boeke, Paolo Mita

**Affiliations:** 1 The Bronx High School of Science, 75 West 205 Street, Bronx, NY 10468 USA; 2 Department of Biochemistry and Molecular Pharmacology, NYU Langone Health, Institute of Systems Genetics (ISG), New York, NY 10016 USA

**Keywords:** Isothermal in vitro assembly, Gibson assembly, Cloning, Deletion, Restriction site

## Abstract

**Background:**

Recombinant DNA technology is today a fundamental tool for virtually all biological research fields. Among the many techniques available for the construction of a “custom DNA” molecule, the isothermal in vitro assembly, or Gibson assembly, allows for an efficient, one-step, scarless recombination-based assembly.

**Results:**

Here, we apply and characterize the use of Gibson assembly for the deletion of DNA sequences around a DNA cut. This method, that we named “Gibson Deletion”, can be used to easily substitute or delete one or more restriction sites within a DNA molecule. We show that Gibson Deletion is a viable method to delete up to 100 nucleotides from the DNA ends of a cleavage site. In addition, we found that Gibson Deletion can be performed using single strand DNA with the same efficiency as using double strand DNA molecules.

**Conclusions:**

Gibson Deletion is a novel, easy and convenient application of isothermal in vitro assembly, that performs with high efficiency and can be implemented for a broad range of applications.

**Electronic supplementary material:**

The online version of this article (10.1186/s12575-018-0068-7) contains supplementary material, which is available to authorized users.

## Background

Recombinant DNA technology has given scientists the ability to edit, join, and delete DNA, driving fundamental discoveries in biology. This technology was initiated by the discovery of DNA ligase and restriction enzymes and it is still applied today for the construction of “custom made” DNA molecules. More recent approaches allow the assembly of DNA molecules without the use of restriction enzymes (PCR mediated assemblies [10, 11], Gateway system developed by Invitrogen) or ligase enzymes (ligation independent cloning or LIC [1]). Moreover, the use of type IIS enzymes enabled the development of seamless cloning strategies able to assemble DNA molecules without the production of “inconvenient” scars left behind by the cloning process itself; the efficient Golden Gate cloning method is an example of these approaches [3].

Isothermal in vitro recombination, also called “Gibson assembly” [4, 5, 7, 13], is a cloning method that assembles DNA molecules with overlapping sequences. The overlapping DNA molecules are joined together by the action of 3 enzymes: a T5 exonuclease that exposes ssDNA complementary sequences able to anneal; a polymerase that fills the gaps between the annealed DNA and a ligase that covalently seals the DNA backbone in vitro [4]. In particular, the one-step isothermal in vitro recombination is an extremely easy, fast, efficient and versatile cloning method that has been applied in many contexts including the assembly of large DNA molecules and the construction of libraries [6, 8]. Our laboratory previously described the use of Gibson cloning for the efficient and convenient mutation of one or multiple sites in a circular DNA [9, 13].

Here, we describe the application of Gibson cloning for the deletion of DNA sequences at the end of linear DNA molecules. The linear DNA can be produced by restriction digestion or can simply be a synthesized DNA molecule (single or double strand). This approach is based on the fact that the homology between the two DNA molecules to be assembled does not necessarily need to be present at the DNA end. We show that if the overlapping sequences of two linear DNA molecules are present within a set range of base pairs away from the DNA ends, the DNA molecules will be assembled and the sequences between the DNA end and the homologous DNA will be deleted. This technique, that we named “Gibson Deletion”, can be conveniently applied for the deletion or substitution of a restriction site or the deletion of DNA sequences around a restriction (or differently produced) cut. We also show that single-stranded DNA oligonucleotides can be successfully used for Gibson Deletion with similar efficiency to that of double-stranded DNAs.

Here, we aim to characterize the use of Gibson Deletion and the limits of its application. We measured the efficiency of Gibson Deletion in several experimental designs demonstrating that Gibson Deletion is an easy and convenient method that expands the application of Gibson cloning.

## Methods

### Plasmid digestion, PCR and bacteria transformation

All digestions were performed for at least 1 h in the presence of the indicated restriction enzyme and calf intestinal alkaline phosphatase (CIP) (NEB cat. #M0290). The linearized plasmid (pUC19) was run on a 1% agarose gel and isolated using Zymo gel recovery kit (Zymo Research cat. #D4002). The isolated DNA was qualitatively estimated running 1 μl of the isolated DNA on an agarose gel and comparing the intensity of the considered band with a known quantity of DNA ladder bands (NEB cat. # N3200).

PCR of the RFP (red fluorescence protein) cassette was performed using primers reported in Additional file 1. GoTaqGreen master mix (Promega, cat. # M7122) was used to amplify the RFP cassette as specified by the company. The PCR products were gel isolated using Zymo gel recovery kit (Zymo Research cat. #D4002) and their amount qualitatively quantified by comparison with the DNA ladder bands (NEB cat. # N3200).

Transformation of Gibson reactions were performed using 10 μl of reaction mix and 90 μl of chemically competent TOP10 cells. The DNA/bacteria were incubated in ice for 30 min, heat shocked at 42 °C for 45 s, and left in ice for 2 min. After heat-shock bacterial cells were grown in 1 ml of LB media for 1 h at 37 °C and 1/10 (100 μl) or 9/10 (900 μl) of bacterial solution was then plated on agarose plates with Carbenicillin selection. Agarose plates with 40 μl of X-gal 20 mg/ml spread on the agarose surface, were used in experiments presented in Fig. 2. Plates were incubated over night at 37 °C and the next day colonies were counted and processed for DNA isolation. DNA was isolated from colonies using Zyppy Plasmid Miniprep Kit (Zymo Research cat. #D4037) as specified by the company.

Sanger sequencing was performed through Genewiz (South Plainfield, NJ).

Oligonucleotides were purchased from IDT, Integrated DNA Technologies (Coralville, Iowa 52,241).

### Gibson assembly and Gibson Deletion reactions

One-step ISO assembly reagents (5X ISO Buffer and Reaction Master Mix) were prepared as previously reported [4]. Briefly, components to be assembled (PCR products, linearized plasmids, single or double strand DNA) were added to 15 μl of Gibson Reaction Master Mix [4] to a final volume of 20 μl. All components are added to an equal molar amount. To this end single strand or annealed DNA pieces were used at a concentration of 100 nM. One-step isothermal assembly/Gibson and Gibson Deletion were performed at 50 °C in a pre-heated heat block for 30 min. 10 μl of reaction were then transformed in 90 μl of TOP10 competent bacteria.

Complementary oligonucleotides purchased from IDT (integrated DNA Technologies) were annealed as follow:

9 μl of 100 μM forward oligonucleotide and 9 μl of 100 μM reverse oligonucleotide were mixed with 2 μl of 10X Annealing buffer (0.5 M Tris-HCl pH 7.4, 0.1 M MgCl_2_). The mixture was then heated for 5 min at 95 °C and cooled off to room temperature in steps of 5 °C 30 s each.

## Results

### Deletion and substitution of restriction sites using “Gibson Deletion”

**Fig. 1:**
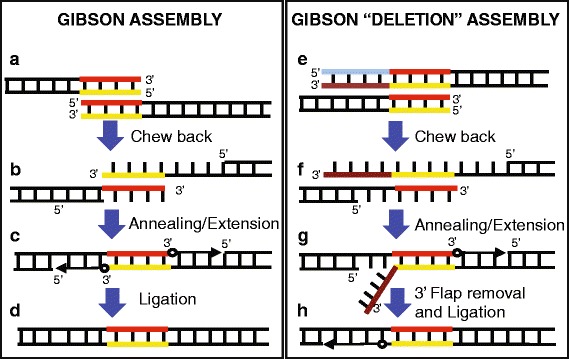
Comparison of classical Gibson and Gibson Deletion assembly. During Gibson reaction two pieces of linear DNA with homologous sequences at their end can recombine into an assembled DNA (left panel). A 5′- > 3′ exonuclease (T5) chews back the DNA ends exposing the homologous sequences (orange and yellow lines) then able to anneal (**a**-**b**). A Phusion DNA polymerase extends the DNA from the 3′ ends (**c**) and a Taq ligase will ligate the nicks (**d**). Gibson Deletion (right panel) is an alternative application of the classical Gibson assembly method that takes advantage of the homologous sequences being present several bases away from the DNA ends (**e**; red and yellow = homologous DNA; blue and brown = non-homologous DNA). This allows for deletion of the non-homologous sequences and assembly of the two linear pieces (**f**-**h**). The created flap-DNA (**g**, brown line) is removed most likely by the 3′- > 5′ exonuclease activity of the Phusion polymerase in the Gibson mix and the nick filled-in and ligated

**Fig. 2:**
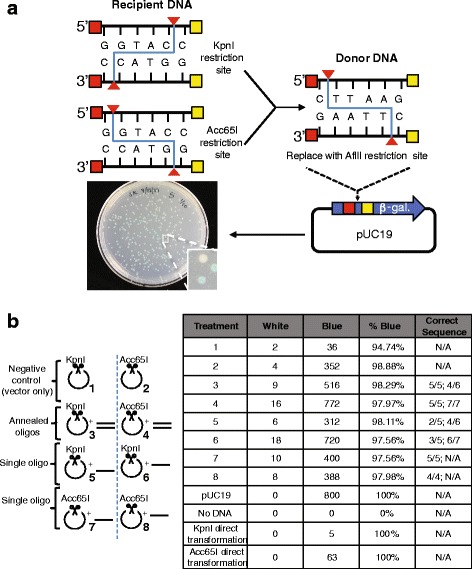
Testing Gibson Deletion. **a** pUC19 plasmid was cut with *Kpn*I or *Acc*65I and these restriction sites present at the 5′ end of the β-galactosidase gene were substituted with an *Afl*II restriction site using Gibson Deletion. The red and yellow boxes represent the homologous sequences of 20 nucleotides each. A picture of blue (likely successful Gibson Deletion) and white (unsuccessful Gibson Deletion) bacteria colonies transformed with Gibson Deletion products is shown. **b** In the left schemes, the DNA added to each Gibson Deletion reactions are shown. The table reports the number of white or blue colonies after transformation and plating of each Gibson Deletion reaction. The number of correct clones after diagnostic cut and Sanger sequencing is reported in the last columns. The results of two independent experiments are shown

Gibson assembly is a powerful cloning technique that allows scarless assembly of pieces of DNA with homologous sequences [4]. Here we challenged this cloning method to assemble DNA pieces with the homologous sequences present at a set number of bases away from the DNA end (Fig. 1). With this approach a flap of non-homologous sequence will be created and removed most likely by the 3′- > 5′ exonuclease activity of the Phusion polymerase present in the Gibson mix (Fig. 1g and h). The gap is then filled in and ligated, resulting in loss of the DNA between the homologous sequences and the end of the DNA. We tested this approach using a small, low complexity plasmid (pUC19) and attempted to delete a restriction site (*Kpn*I/*Acc*65I) and substitute it with a different one (*Afl*II) (Fig. 2). To test whether Gibson Deletion displays an obvious bias towards a specific overhang, we used two enzymes recognizing the same DNA sequence but producing opposite overhangs (neoschizomers): a 5′ overhang with *Acc*65I and a 3′ overhang with *Kpn*I. These enzymes cut the plasmid in the 5′ end of the encoded β-galactosidase enzyme, which we used for the screening of the cloning products. We substituted the *Kpn*I/*Acc*65I site with an *Afl*II site using Gibson Deletion. In particular, we used double strand DNA pieces (annealed oligoes) as well as single strand oligoes as donor DNA for the assembly. The donor DNA contains an AflII restriction site flanked by 20 nucleotides on each side, homologous to pUC19 DNA, flanking the *Kpn*I/*Acc*65I site (Fig. 2a, red and yellow boxes). Both forward and reverse orientations were tested when we used single strand donor DNA (Fig. 2b).

To determine whether the Gibson Deletion occurred correctly, we first performed a qualitative observation of the colonies plated on media supplemented with X-gal, a substrate of β-galactosidase enzyme. Because we substituted one 6 nucleotide restriction site (*Kpn*I/*Acc*65I) with a different 6 nucleotide restriction site (*Afl*II), the β-galactosidase enzyme remained active despite the small nucleotide sequence change. Consequently, colonies expressing a plasmid with correct Gibson Deletion should appear blue, as the β-galactosidase enzyme processes the X-gal substrate (Fig. 2a). We then performed a diagnostic cut of the DNA with *Kpn*I and *Afl*II. To simplify the direct observation of correct DNA bands on a gel we performed *Afl*II or *Kpn*I digest together with *Xmn*I (Additional file 2: Figure S1). A subset of successful products was also Sanger sequenced to verify correct assembly.

Gibson Deletion was performed cutting the vector with *Kpn*I (Fig. 2b, treatment 1, 3, 5, 7) or *Acc*65I (Fig. 2b, treatment 2, 4, 6, 8) in the presence of calf intestinal alkaline phosphatase (CIP). Complementary annealed oligos (Fig. 2b, treatment 3 and 4) or single stranded DNA oligos (Fig. 2b, treatment 5–8) were used as donor DNA. As controls, the cut vectors were used for Gibson Deletion in the absence of donor DNA (Fig. 2b, treatment 3, 5, 7), or the same cut vectors were directly transformed into competent cells without Gibson reaction. This latter treatment is a better control because it allows the measurement of uncut plasmid present in the Gibson reaction.

The results show that Gibson Deletion is a very efficient cloning method, as the colonies were overwhelmingly blue (more than 94% for all conditions; Fig. 2) and the background from the uncut plasmid (direct transformation of cut plasmid) was very low. Moreover, most of the analyzed colonies show a correct substitution of *Kpn*I site with an *Afl*II site (Fig. 2b table and Additional file 2: Figure S1). Furthermore, we observed no preference of 5′ over 3′ overhang for the Gibson Deletion (Fig. 2b, treatments 3, 5, 7 versus 4, 6, 8). Interestingly, single stranded oligos, with no preference towards the forward or reverse strand, are as efficient as double stranded annealed oligoes when used as donor DNA (Fig. 2b, treatment 3,4 versus 5–8).

**Fig. 3:**
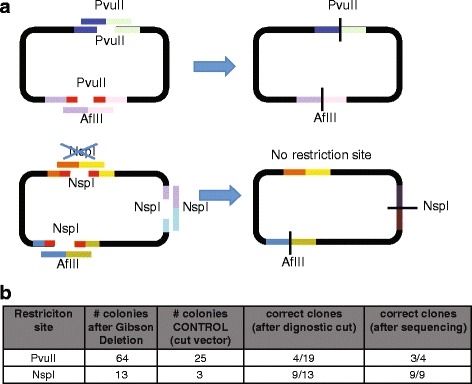
Use of Gibson Deletion to change, delete or maintain a restriction site. **a** Schematic depiction of the two groups of changes introduced in pUC19 using Gibson Deletion: top = making *Pvu*II site unique changing a site into an *Afl*II site and maintain the other *Pvu*II site; bottom = making *Nsp*I site unique deleting a site, maintaining a second site and changing a third *Nsp*I site into an *Afl*II site. **b** Results from the cloning depicted in **a**

Converting a restriction site into a different one is sometime necessary to make a restriction site unique or to create a new unique restriction site suitable for following cloning steps. This seemingly easy tasks, often requires a laborious procedure using standard cloning procedures. We therefore tested our Gibson Deletion approach for these applications using the pUC19 plasmid which contains two *Pvu*II sites and three *Nsp*I sites. Gibson Deletion was performed using single strand oligos that would maintain one *Pvu*II site and mutate the second one into an *Afl*II site (Fig. 3a, top panel). The vector was cut over-night with PvuII in the presence of calf intestinal phosphatase (CIP) and the digestion product simply purified on a PCR purification and concentration column. Gibson Deletion was performed in a single reaction mixing the purified cut vector and the two single strand DNA oligonucleotides with the Gibson reaction mix (Fig. 3b and Additional file 3: Figure S2).

A similar procedure was implemented using a column purified *Nsp*I pUC19 vector and single strand oligoes that would maintain one *Nsp*I site, eliminate a second *Nsp*I site, and mutate the third *Nsp*I site into an *Afl*II site (Fig. 3a bottom panels). To verify correct assembly we isolated the DNA and cut it with *Pvu*II and *Xmn*I or *Afl*II and *Xmn*I for the pUC19 with changed *PvuII* sites (group 1), and with *Nsp*I and *Nde*I or *Afl*II and *Xmn*I for the pUC19 with changed *Nsp*I sites (group 2). Of the 19 colonies analyzed, 4 showed correct assembly for group 1 of the 13 colonies analyzed, 9 showed correct digestion patterns for group 2. We also verified the assemblies through Sanger sequencing that showed mostly correct assemblies (Fig. 3b and Additional file 3: Figure S2).

Overall, we showed that Gibson assembly can also be applied to DNA containing homologous sequences not directly flanking the DNA end. This approach, named “Gibson Deletion”, efficiently produces assembled DNA depleted of the non-homologous regions between the homologous sequences and the DNA end. Gibson Deletion can therefore be used to easily and efficiently change, maintain or eliminate restriction sites.

### Deletion of increasing amount of DNA using Gibson deletion

**Fig. 4:**
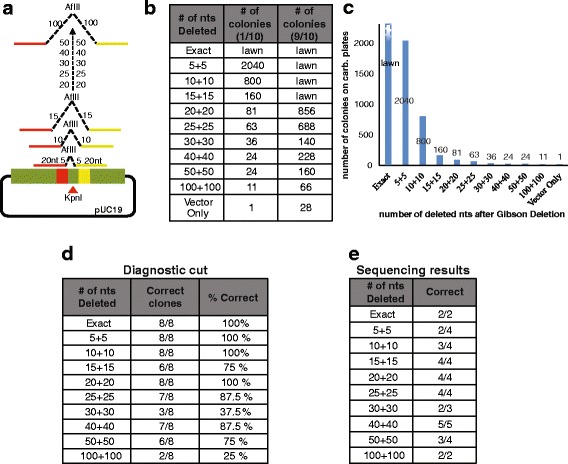
Increasing Deletions Using Gibson reaction. **a** pUC19 plasmid was cut with *Kpn*I and this restriction site was substituted with an *Afl*II site using Gibson Deletion. Single stranded oligos were used as *Afl*II site donor DNA for Gibson reaction. The oligonucleotides used were designed to delete 0, 10 (5 on each side), 20 (10 on each side), 30 (15 on each side), 40 (20 on each side), 50 (25 on each side), 60 (30 on each side), 80 (40 on each side), 100 (50 on each side), and 200 (100 on each side) nucleotides flanking the *Kpn*I/*Afl*II sites. The red and yellow lines and boxes represent homologous sequences of 20 nt on the single stranded oligonucleotides (yellow and red lines) and on the vector (yellow and red boxes). **b**-**c** After Gibson reaction, each sample was transformed and 1/10 or 9/10 of the transformed bacteria were plated on LB plates supplemented with carbenicillin. The table reports the number of growing colonies for each reaction also represented in the bar graph in **c**. **d-e** Number of correct clones after diagnostic cut with *Kpn*I and *Afl*II (**d**) or after Sanger sequence of a subset of samples (**e**)

We showed thatGibson Deletion allows for the deletion/substitution of a restriction site. We then aimed to test how much DNA can be deleted with Gibson Deletion. To this end, nine single stranded donor DNA oligos were designed, with each having homologous sequences with the recipient vector at increasing number of nucleotides away from the DNA end (Fig. 4a). These oligos were designed to delete 0 (exact complementarity to the sequences flanking the *Kpn*I site just substituted with an *Afl*II site), 5, 10, 15, 20, 25, 30, 40, 50, and 100 nucleotides from each end of a *Kpn*I cut (Fig. 4a). Each oligo also substitutes the *Kpn*I site with an *Afl*II site (Fig. 4a). As a control, the cut vector in the absence of donor DNA was used for the Gibson reaction. To perform a first qualitative test of the success of the assembly, we counted the number of colonies that grew on media plates supplemented with the proper selection antibiotics (Carbenicillin). A high number of colonies on the plate indicates a higher likelihood of successful Gibson reaction, as this means that the insert and the vector assembled (Fig. 4b and c). This assumption is corroborated by the limited number of colonies present on the control plate after transformation of only the cut vector (background of uncut plasmid). For a more comprehensive analysis of correct assembly, 8 colonies were picked from each plate, and the DNA was isolated and cut with both *Kpn*I and *Afl*II to determine if the *Afl*II site was correctly substituted to the *Kpn*I site upon successful Gibson reaction (Fig. 4d, e and Additional file 4: Figure S3). Of the successful assemblies, two to five of them were Sanger sequenced to further confirm correct assembly.

The results of the diagnostic cut (Additional file 4: Figure S3) show that, as a greater number of nucleotides are being deleted from both DNA ends of the vector, the Gibson Deletion reaction, as expected, decreases in efficiency, even if a good amount (66 colonies) of colonies could still be found to grow on plates with selection after deleting 200 nucleotides (100 from each side of the new *Afl*II restriction site) (Fig. 4b and c). After diagnostic cut and quantification of *Kpn*I-*Afl*II substitution, deletion of up to 100 nucleotides (50 on each side) showed a success rate of over 75% (excepting the 60 nucleotides deletion that in the presented experiment shows just a 37.5% success rate, most likely due to the small sample size) (Fig. 4d). When deletion of 200 nucleotides was attempted only 2 out of the 8 clones showed *Kpn*I-*Afl*II substitution (25% success rate). This indicates that deletion of more than 100 nucleotides (50 from each side) by Gibson Deletion has a lower efficiency.

DNA that showed *Kpn*I-*Afl*II substitution was Sanger sequenced to confirm that a correct Gibson assembly occurred. Sanger sequencing showed that it was not necessarily true that the more DNA was deleted, the less errors were introduced by the assembly. For the 10 (5 on each side), 20 (10 on each side), 60 (30 on each side) and 100 (50 on each side) nucleotide deletions, at least two of the sequenced clones were found to have the correct sequence. The incorrect assemblies were often incorrect due to an extra deleted nucleotide before or after the inserted *Afl*II site. However, both of the clones sequenced for the more extensive deletion (200 nucleotides around the restriction cut) showed correct assembly (Fig. 4e).

Overall, these results show that hundreds of nucleotides can be easily deleted around a restriction site using single strand DNA in a Gibson Deletion reaction. As expected, a decrease in efficiency is observed in reactions aimed at deleting more than 100 nucleotides.

### Deletion of increasing amount of DNA and simultaneous insertion of complex DNA

**Fig. 5:**
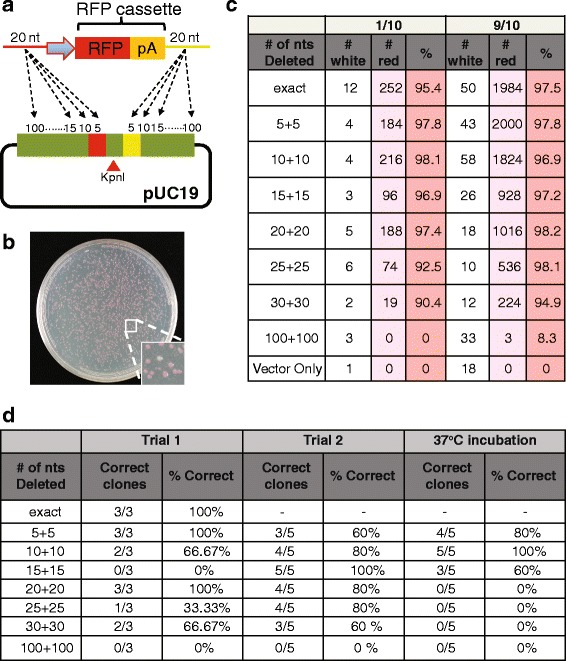
Deletion of increasing amount of DNA and simultaneous insertion of complex DNA. **a** Schematic of the assembly approach followed for the presented experiments. A pUC19 plasmid was cut with *Kpn*I, and a RFP cassette was introduced to the cut vector using Gibson assembly. The different inserts were produced using PCR, each utilizing different primers with different homologous harms to delete increasing amounts of nucleotides around the *Kpn*I site upon Gibson Deletion reaction. The RFP cassettes were designed to have 20 nucleotides of homology with the plasmid (represented by the red and yellow lines and boxes). The RFP primers were designed to delete 0, 10 (5 on each sides), 20 (10 on each sides), 30 (15 on each sides), 40 (20 on each sides), 50 (25 on each sides), 60 (30 on each sides), and 200 (100 on each sides) nucleotides around the *Kpn*I site. **b** The Gibson reactions were transformed and plated on carbenicillin plates (1/10 or 9/10 of the transformation mixture). Colonies expressing DNA that underwent likely successful assembly, appear red due to the expression of the inserted RFP. An incorrect assembly would result in a white colony. **c** The number of red versus white colonies were counted and recorded. **d** Number of correct clones after Sanger sequencing. Gibson Deletion reactions were performed with a 50 °C incubation for 30 min or with a pre-incubation at 37 °C for 30 min before a 50 °C incubation for additional 30 min

To test the efficiency of Gibson Deletion using more complex DNA as donor DNA instead of short oligonucleotides, we performed Gibson assembly using a cassette for RFP (red fluorescent protein) expression. PCR of the RFP cassette was performed using a plasmid template and primers containing homology arms complementary to DNA flanking the *Kpn*I site on the pUC19 plasmid. Several primers were designed to delete increasing amounts of DNA flanking the *Kpn*I site upon Gibson Deletion reaction (Fig. 5a and Additional file 1). Bacterial colonies expressing high amounts of RFP protein (such as for the expression from high copy number plasmids as pUC19) can be easily screened as red colonies on plates; for this reason the RFP cassette was chosen as a simple way to screen colonies expressing plasmids assembled with a correct Gibson reaction (Fig. 5b). Insertion of the RFP cassette and simultaneous deletion of up to 60 nucleotides flanking the *Kpn*I restriction site (30 nucleotides on each side of the *Kpn*I site) using Gibson Deletion produced more than 90% red colonies (Fig. 5c). However, for a deletion of 200 nucleotides (100 on each side) few transformed colonies grew on selection plates and just 8.3% of the colonies were red (Fig. 5c).

Sequencing of the plasmids recovered from the red colonies showed that upon Gibson Deletion most of the DNA underwent correct assembly (Fig. 5d). Deletion of 200 nucleotides around the *Kpn*I sites yielded no correct integration of the RFP cassette. Although we were able to obtain red colonies from this more challenging assembly, Sanger sequencing of the RFP flanking sequences revealed correct deletions only on one side (5′ end or 3′ end) of the RFP and partial or no deletion on the other side of the RFP cassette. Some clones also showed novel insertions between the RFP cassette and the deleted sequences (Additional file 5: Figure S4). Based on these results, we also tested if a 37 °C incubation of the Gibson reaction prior to incubation at 50 °C (see methods) increased the efficiency of Gibson Deletion. The optimal temperature of the T5 exonuclease, necessary to expose the homologous sequences before their annealing, is 37 °C and therefore we hypothesized that a pre-incubation at 37 °C would increase chew back, possibly increasing the success of Gibson Deletion assemblies especially for the reaction with longer sequences to be deleted. Gibson Deletion reactions were therefore incubated for 30 min at 37 °C before incubation at 50 °C for subsequent 30 min. The pre-incubation at 37 °C did not improve Gibson Deletion reaction and it even decreased the number of correct assemblies as determined by DNA sequencing of the obtained plasmids after Gibson Deletion reaction performed in parallel with or without the 37 °C pre-incubation (Fig. 5d).

Overall our results show that Gibson Deletion can be applied for the simultaneous deletion of DNA and assembly of complex DNA with efficiency of assembly decreasing with increasing amounts of deleted DNA.

## Discussion

Recombinant DNA technology has proven very useful in many research fields. Isothermal in vitro recombination, or Gibson assembly, has more recently provided a way to assemble DNA molecules with homologous sequences. Here, we expanded on the application of Gibson assembly in a technique that we named “Gibson Deletion”. This method is based on the fact that Gibson assembly can efficiently take place when the homologous sequences of the DNA pieces that need to be assembled are not present at the DNA ends. We show that in this setting, the DNA between the homologous sequences and the DNA end is deleted. Also, we recorded high efficiency of assembly using single strand DNA as donor DNA. We envisioned this method to be particularly applicable to the deletion and substitution of restriction sites and for the deletion of larger pieces of DNA (up to 100 base pairs) around a restriction site. This approach is particularly effective because it does not require the use of polymerase chain reaction (PCR) for the simple deletion or substitution of small DNA stretches. We applied Gibson Deletion for easy deletion/substitution of multiple *PvuII* and *NspI* restriction sites in pUC19 plasmid. This approach produced new restriction sites, and rendered both PvuII and NspI unique in their respective trials.

We also show that Gibson Deletion can be applied using DNA more complex than simple oligonucleotides. We used a PCR amplified RFP cassette and insert it in pUC19 plasmid, simultaneously deleting up to 100 nucleotides (50 on each side of the restriction cut) around a restriction site. We can envision several applications for this approach including the construction of large plasmid collections of mutants or variations of a specific sequence. For instance, a unique restriction site can be cloned in the desired position and subsequently eliminated during Gibson assembly using inserts with homologous arms that do not include the sequence of the new restriction site, just used as intermediate cloning tool. For instance, multiple tags can be easily cloned at the 5′- or 3′- end of a coding gene to test their impact on the encoded protein. Also, the generation of multiple mutations at the desired location in a vector can be easily achieved by Gibson Deletion with the simple use of a single strand DNA library of mutated sequences and a vector with a restriction site in the desired position.

Previous work [12] showed the application of Gibson assembly using homologous sequences away from the DNA ends created by a CRISPR/Cas9 in vitro cut. Wang et al. [12], showed that Gibson Deletion can be applied to any DNA end, created by DNA synthesis, by restriction digest or by CRISPR/Cas9 cut. Here we better describe and characterize how this particular use of Gibson assembly performs in different settings and conditions.

We observed a large decrease of efficiency of Gibson Deletion as the number of nucleotides deleted increased, especially using complex DNA. In particular, we were not able to retrieve correct plasmids with an inserted RFP cassette and simultaneous deletion of 200 nucleotides around a *Kpn*I site (Fig. 5). Sequencing of the incorrect clones (Additional file 5: Fig. S4) shows that if RFP assembly occurred, only partial deletions were achieved, mostly on one side (5′ or 3′) of the RFP. Most likely micro-homology between the donor DNA end and any DNA sequence of the recipient DNA mediates these spurious partial assemblies. The longer the distance between the homologous sequences shared by the donor and acceptor DNA and the DNA ends is, the higher the chance of having micro-homology mediated assembly will be. These spurious assemblies can be facilitated on one side by the correct annealing and assembly on the opposite side of the donor DNA.

It is worth discussing the choice of relevant controls in Gibson reactions. In our work we used two kind of controls: the cut vector used for Gibson Deletion in the absence of donor DNA, or the same cut vector directly transformed into competent cells without Gibson reaction. In our experience presence of colonies after transformation of both controls is not usually correlated with the success of the Gibson. Possible recombination events within the cut vector sequence favored by the absence of donor DNA may partially explain why the described controls are not always indicative of correct Gibson reaction. On the other hand, a low number or an absence of colonies in the controls will most likely correlate with a successful Gibson in the experimental reaction. In light of our experience, we always performed at least one of the control reactions in Gibson assembly experiments. We use the number of colonies in the controls to determine the number of colonies from the experimental reaction that will be analyzed further; we collect fewer colonies if controls show few or no colonies while a higher number of colonies will be analyzed if a greater background is observed. In general, upon Gibson assembly, colonies from experimental reactions should always be analyzed even if control reactions result in a higher number of colonies.

Future experiments will need to be conducted for the optimization of Gibson Deletion. Different parameters within the Gibson mix itself could be altered. For example, different amounts of the enzymes (exonuclease, polymerase, and/or ligase) may be used to determine the optimal ratio of enzymes that would yield more efficient Gibson Deletion reactions. In addition, different enzymes may also be tested. For instance, a polymerase other than Phusion with a more efficient 3′-5′ exonuclease activity may render the Gibson Deletion reaction more efficient. It needs to be mention that, despite the hypothesized role of the 3′-5′ exonuclease activity of the Phusion polymerase to resolve the DNA Flap structure formed during Gibson Deletion, it is formally possible that DNA damage repair mechanisms present in the competent bacteria cells are responsible for this process. Flap endonuclease such as FEN1 [2], may cleave the 5′ flaps not eliminated in vitro during Gibson Deletion reaction, and repair mechanism of the cell may fill in and ligate the gap. The assembly reactions, possibly happening in competent cells after transformation, would justify the fact that single stranded DNA can be efficiently used for Gibson reactions. The present model of Gibson assembly cannot completely explain this finding. A clearer view of the important steps of Gibson Deletion needs to be gained to improve the technique and its efficiency.

## Conclusions

Overall the results presented here establish Gibson Deletion as a useful and novel application of Gibson assembly expanding the already broad application of isothermal in vitro recombination approaches.

## Additional files

Additional file 1: The sequences of the oligonucleotides used in this study are reported. (XLSX 58 kb)

Additional file 2: Figure S1. Diagnostic cut related to Fig. 2. DNA isolated from blue clones was cut with *Kpn*I + *Xmn*I or *Afl*II + *Xmn*I and run on an agarose gel. The expected correct band pattern is reported in the inset on the right. A scheme of the DNA components used in each assembly reaction is reported on top of the corresponding clones. Uncut pUC19 DNA is run on the last lane of the top two gels. DNA resulting from an incorrect assembly is labelled with a X on the bottom of the corresponding clone. (PDF 13592 kb)

Additional file 3: Figure S2. Diagnostic cut related to Fig. 3. DNA isolated from clones that grew on selection plates was cut with the indicated enzymes. Group 1 cloning is depicted in Fig. 3a top panels and Group 2 cloning is depicted in Fig. 3a bottom panels. Images of simulated agarose gels are presented on the left of each cloning group. Red arrowheads indicate the clones with DNA that underwent successful/correct Gibson Deletion reaction. (PDF 3775 kb)

Additional file 4: Figure S3. Diagnostic cut related to Fig. 4. DNA isolated from blue clones was cut with *Kpn*I or *Afl*II and run on an agarose gel. The expected correct band pattern is reported in the inset on the right. The expected number of nucleotides deleted by Gibson Deletion (smaller deletions on top and larger deletions on the bottom) is reported on top of each group of lanes. Uncut, *Afl*II or *Kpn*I cut pUC19 are run on the left lanes of each gel as reported. Each clone’s DNA was cut with *Afl*II or *Kpn*I and the two digestions run on consecutive lanes. DNA resulting from an incorrect assembly is labelled with a X on the bottom of the two lanes corresponding to the incorrect clone. (PDF 15481 kb)

Additional file 5: Figure S4. Sequences of the incorrect Gibson Deletion of 200 nt related to Fig. 5. Sequences of the DNA flanking the RFP cassette (red box) cloned in pUC19 using Gibson Deletion. The RFP cassette was PCR amplified using primers with homology arms aimed to delete 200 nts (100 nucleotides from each side of a *Kpn*I cut) from the pUC19 plasmids (Fig. 5). All the clones picked and sequences had incorrect assembly and are presented here together with the predicted sequence on top (correct). Underlined sequences are part of the RFP cassette; red sequences are sequences that are deleted after assembly using Gibson Deletion; sequences in bold are insertions obtained after assembly. (PDF 1358 kb)

## Abbreviations

CIP: Calf intestinal alkaline phosphatase
PCR: Polymerase chain reaction
RFP: Red fluorescent protein
X-gal: 5-bromo-4-chloro-3-indolyl-β-D-galactopyranoside
β-gal: β galactosidase
