# Supplementary figures and images for: Gibson Deletion: a novel application of isothermal in vitro recombination
Source: Biol Proced Online. 2018 Jan 19;20:2. doi: 10.1186/s12575-018-0068-7 (PMC5774033; doi:10.1186/s12575-018-0068-7)

# Supplementary Figure 1

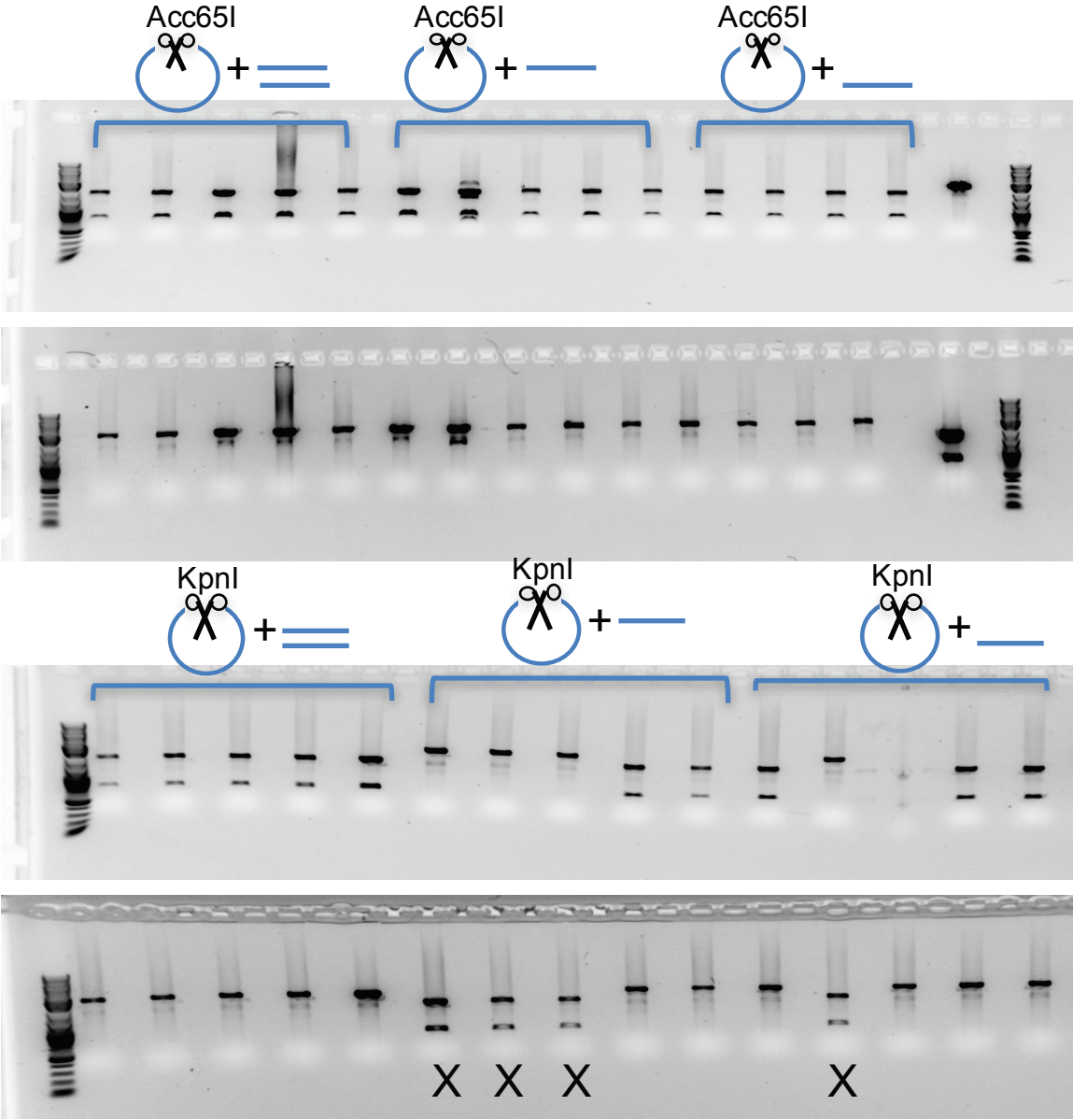

CUT WITH:

AflIII+XmnI

KpnI+XmnI

AflIII+XmnI

KpnI+XmnI

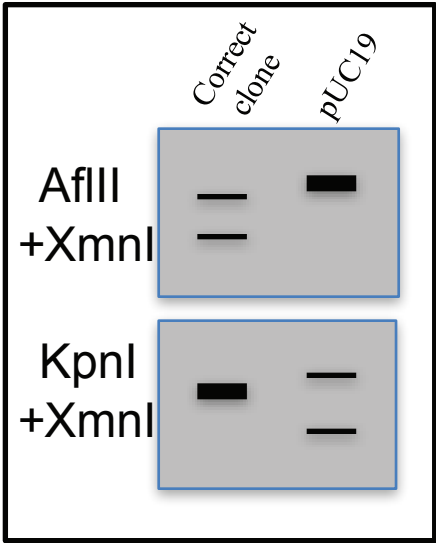

Supplement: Supplementary file 2 — Diagnostic cut related to Fig. 2. DNA isolated from blue clones was cut with KpnI + XmnI or AflII + XmnI and run on an agarose gel. The expected correct band pattern is reported in the inset on the right. A scheme of the DNA components used in each assembly reaction is reported on top of the corresponding clones. Uncut pUC19 DNA is run on the last lane of the top two gels. DNA resulting from an incorrect assembly is labelled with a X on the bottom of the corresponding clone. (PDF 13592 kb) [file 12575_2018_68_MOESM2_ESM.pdf]

# Supplementary Figure 2

Group 1

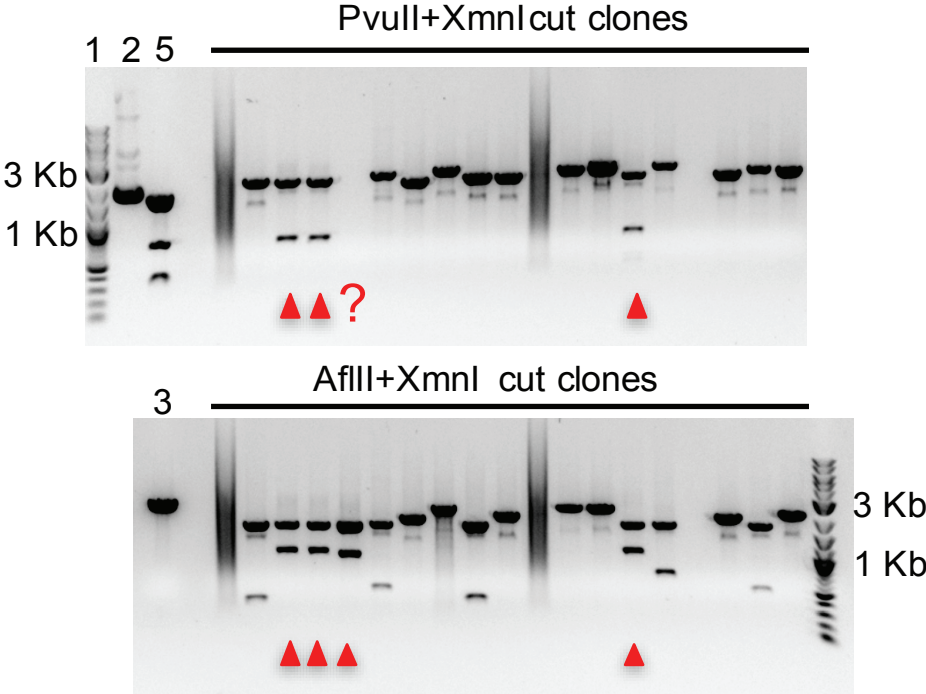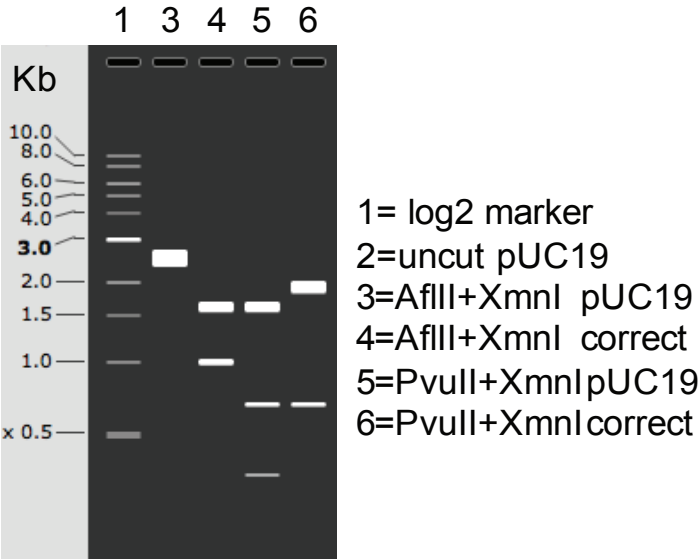

Group 2

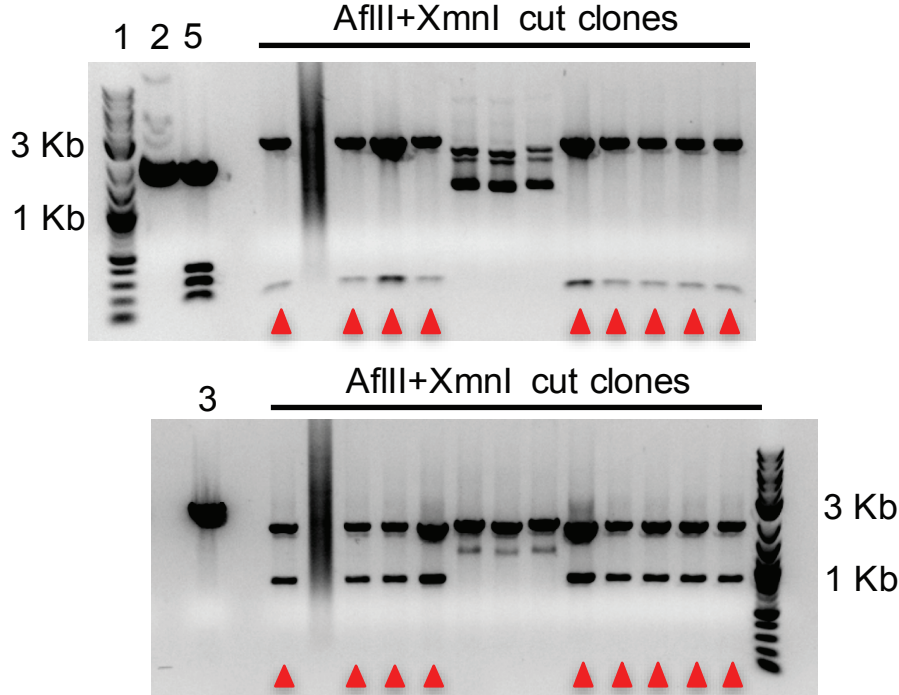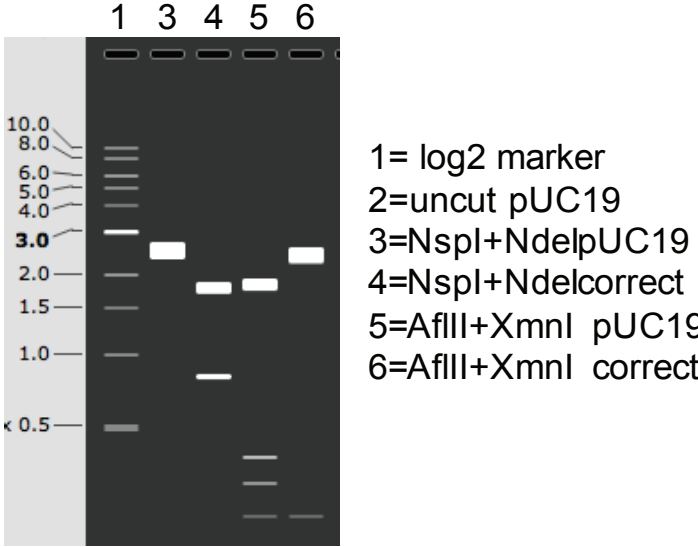

Supplement: Supplementary file 3 — Diagnostic cut related to Fig. 3. DNA isolated from clones that grew on selection plates was cut with the indicated enzymes. Group 1 cloning is depicted in Fig. 3a top panels and Group 2 cloning is depicted in Fig. 3a bottom panels. Images of simulated agarose gels are presented on the left of each cloning group. Red arrowheads indicate the clones with DNA that underwent successful/correct Gibson Deletion reaction. (PDF 3775 kb) [file 12575_2018_68_MOESM3_ESM.pdf]

# Supplementary Figure 3

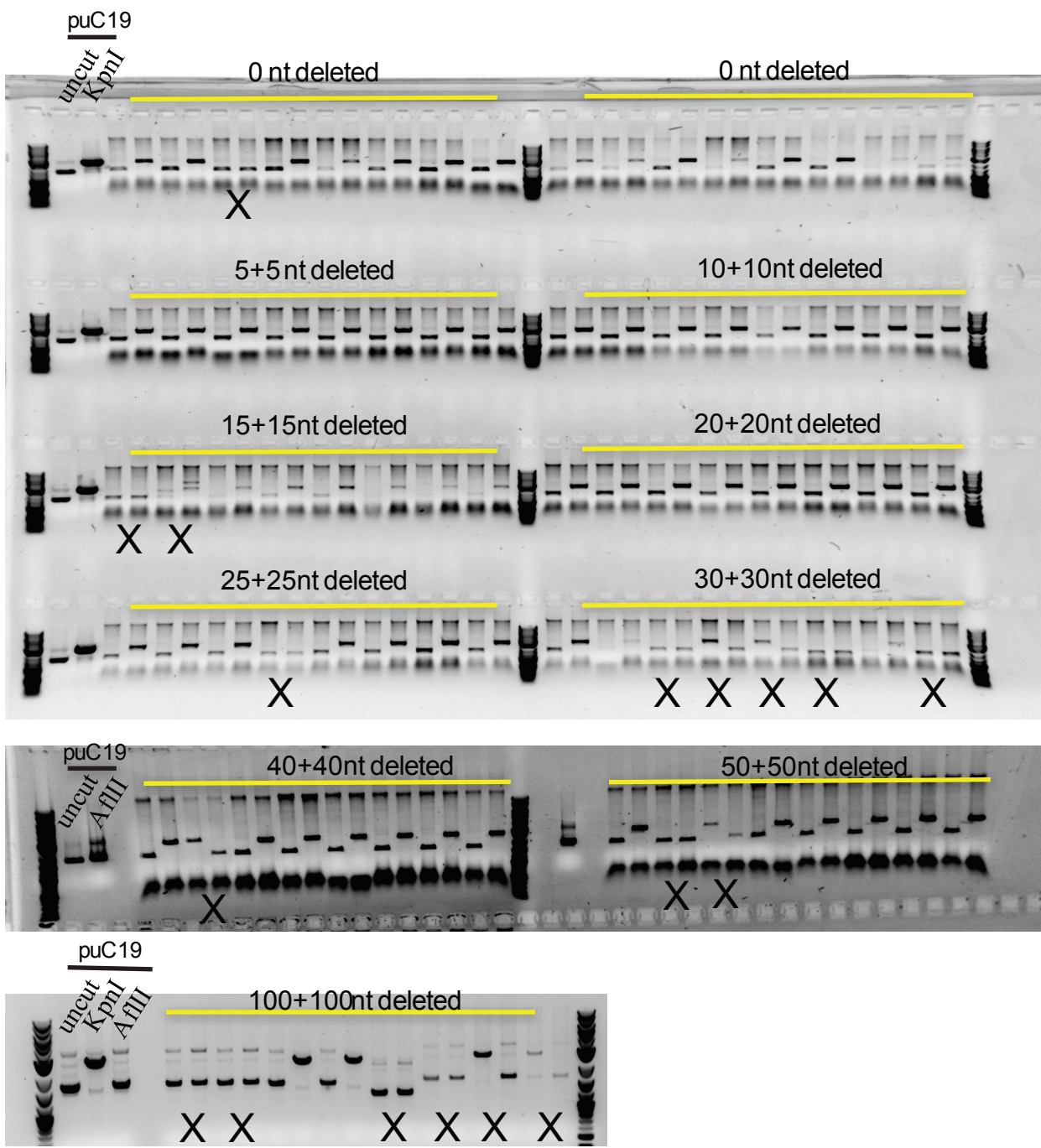

Smaller deletion

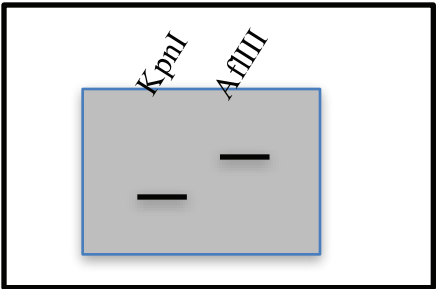

Bigger deletion

Supplement: Supplementary file 4 — Diagnostic cut related to Fig. 4. DNA isolated from blue clones was cut with KpnI or AflII and run on an agarose gel. The expected correct band pattern is reported in the inset on the right. The expected number of nucleotides deleted by Gibson Deletion (smaller deletions on top and larger deletions on the bottom) is reported on top of each group of lanes. Uncut, AflII or KpnI cut pUC19 are run on the left lanes of each gel as reported. Each clone’s DNA was cut with AflII or KpnI and the two digestions run on consecutive lanes. DNA resulting from an incorrect assembly is labelled with a X on the bottom of the two lanes corresponding to the incorrect clone. (PDF 15481 kb) [file 12575_2018_68_MOESM4_ESM.pdf]
