# Supplementary material for: Gibson Deletion: a novel application of isothermal in vitro recombination
Source: Biol Proced Online. 2018 Jan 19;20:2. doi: 10.1186/s12575-018-0068-7 (PMC5774033; doi:10.1186/s12575-018-0068-7)
Supplement: Supplementary file 5 — Sequences of the incorrect Gibson Deletion of 200 nt related to Fig. 5. Sequences of the DNA flanking the RFP cassette (red box) cloned in pUC19 using Gibson Deletion. The RFP cassette was PCR amplified using primers with homology arms aimed to delete 200 nts (100 nucleotides from each side of a KpnI cut) from the pUC19 plasmids (Fig. 5). All the clones picked and sequences had incorrect assembly and are presented here together with the predicted sequence on top (correct). Underlined sequences are part of the RFP cassette; red sequences are sequences that are deleted after assembly using Gibson Deletion; sequences in bold are insertions obtained after assembly. (PDF 1358 kb) [file 12575_2018_68_MOESM5_ESM.pdf]

Supplementary Figure 4

Correct

CTTCGCTATTACGCCAGCTGGCGAAAGGGGGATGTGCTGCAAGGCGAT
TAAGTTGGGTAACGCCAGGGTTTTCCCAGTCACGACGTTGTAAAACGAC
GGCCAGTGAATTCGAGCTCGGTACGAATTCGCGGCCGCTTCTAG

RFP

ACTAGTAGCGGCCGCTGCAGCCGGGGATCCTCTAGAGTCGACCTGCAG
GCATGCAAGCTTGGCGTAATCATGGTCATAGCTGTTTCCTGTGTGAAATT
GTTATCCGCTCACAATTCCACACAACATACGAGCCGGAAGCATA

Trial 1

CTTCGCTATTACGCCAGCTGGCGAAAGGGGGATGTGCTGCAAGGCGAT
TAAGTTGGGTAACGCCAGGGTTTTCCCAGTCACGACGTTGTAAAACGAC
GGCCAGTGAATTCGAGCTCGGTACGAATTCGCGGCCGCTTCTAG

RFP

ACTAGTAGCGGCCGCTGCAG**TCAGACGGTCTCATTTTG**CCGGGGATCC
ACTAGAGTCGACCTGCAGGCATGCAAGCTTGGCGTAATCATGGTCATAG
CTGNTCCTTGNGTGAAATTGTTATCCGCTCACAATTCCACACCAACATAC
GAGCCGGAAGCA

CTTCGCTATTACGCCAGCTGGCGAAAGGGGGATGTGCTGCAAGGCGAT
TAAGTTGGGTAACGCCAGGGTTTTCCCAGTCACGACGTTGTAAAACGAC
GGCCAGTGAATTCGAGCTCGGTACGAATTCGCGGCCGCTTCTAG

RFP

ACTAGTAGCGGCCGCTGCAGCCGGGGATCCTCTAGAGTCGACCTGCAG
GCATGCAAGCTTGGCGTAATCATGGTCATAGCTGTTTCCTGTGTGAAATT
GTTATCCGCTCACAATTCCACACAACATACGAGCCGGAAGCATA

CTTCGCTATTACGCCAGCTGGCGAAAGGGGGATGTGCTGCAAGGCGATT
AAGTTGGGTAACGCCAGGGTTTTCCCAGTCACGACGTTGTAAAACGACG
GCCAG**G**TGAATTCGAGCTCGGTACGAATTCGCGGCCGCTTCTAG

RFP

ACTAGTAGCGGCCGCTGCAGC**ANNA**CGGGGATCCTCTAGAGTCGACCT
GCAGGCATGCAAGCTTGGCGTAATCATGGTCATAGCTGTTTCCTGTGTG
AAATTGTTATCCGCTCACAATTCCACACAACATACGAGCCGGAAGCATA

Trial 2

CTTCGCTATTACGCCAGCTGGCGAAAGGGGGATGTGCTGCAAGGCGAT
TAAGTTGGGTAACGCCAGGGTTTTCCCAGTCACGACGTTGTAAAACGA
CGGCCAGTGAATTCGAGCTCGGTA**T**GAATTCGCGGCCGCTTCTAG

RFP

ACTAGTAGCGGCCGCTGCAGCCGGGGATCCTCTAGAGTCGACCTGCAG
GCATGCAAGCTTGGCGTAATCATGGTCATAGCTGTTTCCTGTGTGAAATT
GTTATCCGCTCACAATTCCACACAACATACGAGCCGGAAGCATA

CTTCGCTATTACGCCAGCTGGCGAAAGGGGGATGTGCTGCAAGGCGAT
TAAGTTGGGTAACGCCAGGGTTTTCCCAGTCACGACGTTGTAAAACGAC
GGCCAGTGAATTCGAGCTCGGTATGAATTCGCGGCCGCTTCTAG

RFP

ACTAGTAGCGGCCGCTGCAGCCGGGGATCCTCTAGAGTCGACCTGCAG
GCATGCAAGCTTGGCGTAATCATGGTCATAGCTGTTTCCTGTGTGAAATT
GTTATCCGCTCACAATTCCACACAACATACGAGCCGGAAGCATA

CTTCGCTATTACGCCAGCTGGCGAAAGGGGGATGTGCTGCAAGGCGAT
TAAGTTGGGTAACGCCAGGGTTTTCCCAGTCACGACGTTGTAAAACGAC
GGCCAGTGAATTCGAGCTCGGTATGAATTCGCGGCCGCTTCTAG

RFP

ACTAGTAGCGGCCGCTGCAGCCGGGGATCCTCTAGAGTCGACCTGCAG
GCATGCAAGCTTGGCGTAATCATGGTCATAGCTGTTTCCTGTGTGAAATT
GTTATCCGCTCACAATTCCACACAACATACGAGCCGGAAGCATA

CTTCGCTATTACGCCAGCTGGCGAAAGGGGGATGTGCTGCAAGGCGAT
TAAGTTGGGTAACGCCAGGGTTTTCCCAGTCACGACGTTGTAAAACGAC
GGCCAGTGAATTCGAGCTCGGTA**T**GAATTCGCGGCCGCTTCTAG

RFP

ACTAGTAGCGGCCGCTGCAGCCGGGGATCCTCTAGAGTCGACCTGCAG
GCATGCAAGCTTGGCGTAATCATGGTCATAGCTGTTTCCTGTGTGAAATT
GTTATCCGCTCACAATTCCACACAACATACGAGCCGGAAGCATA

CTTCGCTATTACGCCAGCTGGCGAAAGGGGGATGTGCTGCAAGGCGAT
TAAGTTGGGTAACGCCAGGGTTTTCCCAGTCACGACGTTGTAAAACGAC
GGCCAGTGAATTCGAGCTCGGTATGAATTCGCGGCCGCTTCTAG

RFP

ACTAGTAGCGGCCGCTGCAG**ATGCCACCTTGGTGTAATCGTGGGCATANC**
TGTTTGCNGTGNGAAAGGTCTAACCCGGGGATCCTCTAGAGTCGACCTGC
AGGCATGCAAGCTTGGCGTAATCATGGTCATAGCTGTTTCCTGTGTGAAATT
GTTATCCGCTCACAATTCCACACAACATACGAGCCGGAAGCATA
